# Geographical Characterization of Olive Oils from the North Aegean Region Based on the Analysis of Biophenols with UHPLC-QTOF-MS

**DOI:** 10.3390/foods10092102

**Published:** 2021-09-06

**Authors:** Evangelia Kritikou, Natasa P. Kalogiouri, Marios Kostakis, Dimitrios-Christos Kanakis, Ioannis Martakos, Constantina Lazarou, Michalis Pentogennis, Nikolaos S. Thomaidis

**Affiliations:** Laboratory of Analytical Chemistry, Department of Chemistry, National and Kapodistrian University of Athens, Panepistimiopolis Zografou, 15771 Athens, Greece; evkritik@chem.uoa.gr (E.K.); kalogiourin@chem.uoa.gr (N.P.K.); makostak@chem.uoa.gr (M.K.); kanakisdim@chem.uoa.gr (D.-C.K.); johnmrtk@chem.uoa.gr (I.M.); clazarou@chem.uoa.gr (C.L.); pentogmi@otenet.gr (M.P.)

**Keywords:** bioactive compounds, phenolic profile, EVOO, Greek, geographical origin, Lesvos

## Abstract

Olive oil is famous due to the nutritional properties and beneficial health effects. The exceptional properties of virgin (VOO) and extra virgin olive oil (EVOO) are credited to the bioactive constituents of their polar fraction, the phenolic compounds. The concentration and composition of biophenols can be influenced by the geographical origin, the cultivar, as well as several agronomic and technological parameters. In this study, an ultra-high-performance liquid chromatography coupled to quadrupole-time of flight tandem mass spectrometry (UHPLC-QTOF-MS) method was used to determine biophenols in Greek EVOOs from five islands originating from the North Aegean Region (Chios, Fournoi, Ikaria, Lesvos, and Samos) through target and suspect screening. In total, 14 suspect and 5 target compounds were determined in the analyzed EVOOs. The quantitative and semiquantitative results were compared to investigate discriminations between different regions. Significant differences were found between the islands based on the overall phenolic content and the concentration levels of individual compounds, as well. In the case of Lesvos, the territory was separated in subdivisions (zones), and each zone was studied individually.

## 1. Introduction

Virgin olive oil (VOO) consists mainly of triglycerides, fatty acids, phospholipids, and sterol esters (>98%). The remaining 1–2% of the VOO composition constitutes the unsaponifiable fraction and contains more than 200 compounds from different chemical categories such as hydrocarbons, sterols, aliphatic and triterpenic alcohols, pigments, tocopherols, phenols, and volatile compounds [1,2]. Among other edible oils, extra virgin olive oil (EVOO) is unique because of the presence of phenolic compounds, commonly called biophenols. Due to their chemical diversity, biophenols are classified into five main classes: (a) phenolic acids; (b) phenolic alcohols; (c) secoiridoids; (d) flavonoids; (e) lignans [3,4].

It is important to highlight that the biophenols, and mainly the secoiridoids such as oleuropein aglycone and oleocanthal, are responsible for the organoleptic characteristics of EVOOs, specifically, the bitter and pungent taste. Moreover, these compounds relate to the oxidative stability of VOO and contribute to its long shelf life as compared to other edible vegetable oils [1,5,6,7,8,9]. Another reason for which the biophenols are great of interest is their biological and pharmacological properties. Phenolics are the main antioxidants in EVOOs [10]. Compounds of EVOO with strong antioxidant activity are hydroxytyrosol and oleocanthal [9,11]. Other biophenols that act against oxidative stress are luteolin, tyrosol, vanillin [12], acetoxypinoresinol, and pinoresinol [11]. Except for their antioxidant activity, biophenols exhibit anti-inflammatory, antiviral, and antimicrobial properties, and contribute to the protection against diseases such as cancer, obesity, diabetes, kidney, neurodegenerative, and cardiovascular diseases, as well [2,9,13,14,15].

Biophenols can be influenced by many parameters, for instance, olive variety [16,17,18], agronomic factors [19,20], cultivation practices [21], as well as olive oil processing such as malaxation conditions and type of extraction system [5,22,23]. The phenolic profile and concentration levels of individual biophenols are strongly related to the geographical location where the *Olea europaea* trees are cultivated [7,24,25,26,27]. Therefore, it is crucial to assess the phenolic profile of EVOOs originating from different regions and distinguish differences that could lead to their classification in relation to the geographical area. In addition, even though there are many research publications about the correlation between the biophenols and the geographical origin for the four of the five major olive oil-producing countries, specifically, Spain [25,28,29], Italy [30,31,32,33], Tunisia [7,24,26,27,34], and Turkey [35,36], the literature about Greek EVOOs is scarce. To the best of our knowledge, most of the works have examined Koroneiki mainly from Peloponnese and Crete [37,38], while there are no available data for Koroneiki, Kolovi, Adramitiani, Throumpa, and other varieties originating from the North Aegean Region.

For the qualitative and quantitative determination of biophenols in VOOs, the majority of the analytical methods involve the use of high-performance liquid chromatography (HPLC) coupled to a diode array detector (DAD) [4,39,40,41] or mass spectrometer (MS) [2,41,42,43,44,45]. High-resolution mass spectrometry (HRMS), due to its high mass accuracy and sensitivity [46], constitutes a powerful analytical tool for the determination of the biophenols in Greek VOOs/EVOOs [42,47], and different organs of the olive tree such as Greek olives [48], olive leaves, and drupes [49], using target and nontarget screening workflows.

The main objective of this work was to analyze the phenolic fingerprint of Greek olive oils from different areas in the Northeastern Aegean Region in order to study differences related to the geographical origin. For this purpose, 452 EVOOs from the islands of Lesvos, Samos, Chios, Ikaria, and Fournoi were analyzed with ultra-high performance liquid chromatography coupled to quadrupole-time of flight tandem mass spectrometry (UHPLC-QTOF-MS) using target and suspect screening strategies. The identified compounds were quantified and semiquantified, and the data were evaluated using descriptive statistics and analysis of variance (ANOVA). The phenolic fingerprints were compared between: (a) the islands; (b) seven geographical locations of the island of Lesvos. The comparison was based on the bioactive content and the four most abundant compounds (oleuropein aglycone, ligstroside aglycone, oleacein, and oleocanthal) in samples. Moreover, the study focused on differences in the phenolic profile of EVOOs based on the percentage of each compound in relation to the phenolic content.

## 2. Materials and Methods

### 2.1. Chemicals and Standards

Ultrapure water was obtained through a purification system (Millipore Direct-Q UV, Bedford, MA, USA). Formic acid, of LC-MS grade, and ammonium acetate ≥99% were obtained from Fluka (Buchs, Switzerland). Sodium hydroxide >99% and methanol, of LC-MS grade, were acquired from Merck (Darmstadt, Germany). 2-propanol was acquired from Fisher Scientific (Geel, Belgium).

All standards had an analytical grade ≥95%. Epicatechin, ferulic acid, gallic acid, homovanillic acid, oleuropein, pinoresinol, p-coumaric acid, and syringaldehyde were purchased from Sigma-Aldrich (Steinheim, Germany). Apigenin, caffeic acid, tyrosol, and vanillin were acquired from Alfa Aesar (Karlsruhe, Germany). Luteolin and hydroxytyrosol were purchased from Santa Cruz Biotechnologies (Heidelberg, Germany). Syringic acid was obtained from Extrasynthèse (Genay, France). Stock solutions for each analyte (1000 mg/L) were dissolved in methanol and stored at −20 °C. Mixed standard working solutions were prepared every laboratory day diluting the stock solutions with water:methanol (20:80, *v*/*v*).

### 2.2. Instrumentation

A UHPLC system consisting of an HPG 3400 pump (Dionex UltiMate 3000 RSLC, Thermo Fisher Scientific, Germany) in combination with a QTOF mass spectrometer (Maxis Impact, Bruker Daltonics, Bremen, Germany) was used. An Acclaim RSLC C18 column (2.1 × 100 mm, 2.2 μm) obtained from Thermo Fisher Scientific (Dreieich, Germany) was utilized at 30 °C for the analysis, equipped with an ACQUITY UPLC BEH C18 1.7 μm, VanGuard precolumn (Waters, Dublin, Ireland). The mobile phase consisted of: (a) 5 mM of ammonium acetate in methanol, (b) 5 mM of ammonium acetate in water:methanol (90:10, *v*/*v*). The gradient program lasting 20 min is presented in Supplementary Material Table S1.

As for the MS parameters, the electrospray negative ionization (ESI –) mode was applied. The capillary voltage was set at 3500 V, the nebulizer pressure was 2 bar (N_2_), the end plate offset was set at 500 V, the drying gas flow rate was equal to 8 L/min (N_2_), and the drying temperature was set at 200 °C. A cluster solution consisting of 10 mM of sodium formate in a mixture of 2-propanol:water (1:1, *v*/*v*) was used for external calibration. Additionally, for internal calibration, the cluster solution was injected at the beginning of each run, in a segment between 0.1 and 0.25 min. Full scan mass spectra, with a scan rate of 2 Hz, were recorded in the range between 50 and 1000 *m*/*z*. MS/MS data were collected using the data-dependent acquisition mode based on the fragmentation of the five most abundant precursor ions per scan (AutoMS, otofControl, Bruker Daltonics). The instrument provided a typical resolving power (full-width at half-maximum, FWHM) between 36,000 and 40,000 at *m*/*z* 226.1593, 430.9137, and 702.8636.

### 2.3. Samples

**Figure 1:**
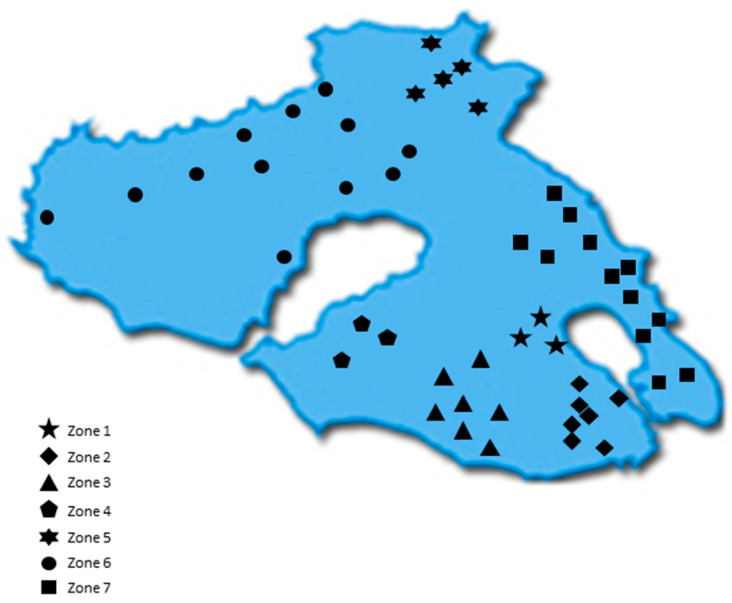
Map of Lesvos indicating the geographical areas (zones) of the island.

All samples were acquired from the Northeastern Aegean islands of Chios, Fournoi, Ikaria, Lesvos, and Samos during the harvesting period 2017–2018. Overall, 452 Greek EVOOs were gathered. Table S2 summarizes the main olive tree varieties and the number of EVOO samples for each island. In the case of Lesvos, due to the size of the island and increased number of samples, the island was divided into 7 geographical zones (Figure 1) based on the location where the olive trees were cultivated. Table S3 presents the number of samples corresponding to each zone. After collection, the samples were stored at 4 °C throughout the study.

### 2.4. Sample Preparation and Quality Control

A validated in-house liquid–liquid extraction (LLE) method [50] was used to isolate the biophenols from the EVOOs. First, 0.5 g of the sample was weighed in an Eppendorf tube and spiked with internal standard (syringaldehyde) at a concentration of 1.3 mg/L. For the extraction, 0.5 mL of water:methanol (20:80, *v*/*v*) was used. Then, the mixture was vortexed for 2 min and centrifuged for 5 min at 13,400 rpm. In the next step, the upper phase was filtered through a 0.22 μm filter (CHROMAFIL^®^ RC, Macherey-Nagel, Düren, Germany). The extract was kept at −80 °C prior to analysis. Then, 5 μL of this solution was injected into the analytical system.

In order to ensure the accuracy and reliability of the results, quality control (QC) was performed using a mixture of the standards apigenin, vanillin, oleuropein, tyrosol, and hydroxytyrosol at a concentration of 1 mg/L. At the beginning of the analysis, the mixture of these compounds was injected six times for conditioning of the equipment, and then it was injected every ten injections during the analysis. The results of QC, specifically, the % relative standard deviation (%RSD) for the peak areas, retention time (t_R_), and Δ*m* errors (*n* = 12) of these five analytes for one laboratory day are presented in Table S4, proving the good performance of the instrument during the analysis. A procedural blank was also prepared in order to detect any potential contamination.

### 2.5. Target and Suspect Screening—Data Processing

A large number of biophenols were identified in EVOO and other organs of *Olea europaea* such as olive leaves, drupes, stems, and roots [2,12,25,51,52,53,54,55,56]. In this work, the study focused on the biophenols identified in VOO in the previous work of our group [42]. For this purpose, a target list of 14 biophenols and a suspect list of 15 bioactive compounds were used and can be found in Tables S5 and S6, respectively. The tables include information for the compounds such as the molecular formula, *m*/*z*, t_R_, and MS/MS fragments, as referred by Kalogiouri et al. [42].

The data were processed via Bruker software, TASQ 1.4 and DataAnalysis 4.4. The determination of the compounds was based on mass accuracy, retention time, isotopic pattern, and MS/MS fragmentation. In particular, extracted ion chromatograms (EICs) were acquired, and the following parameters were applied in the case of target screening: mass accuracy ≤ 2.5 mDa, tolerance of the retention time below ±0.2 min, isotopic fit ≤ 100 mSigma, ion intensity ≥ 500, peak area ≥ 2000, and signal-to-noise (S/N) ≤ 3. In the case of suspect screening, the parameters applied were: mass accuracy ≤ 2.5 mDa, ion intensity ≥ 800, peak area ≥ 3000, and isotopic fit ≤ 100 mSigma. The experimental t_R_ of each analyte was compared with the t_R_ found in VOO samples (Table S6) according to Kalogiouri et al. [42], and the difference was set not to exceed ±0.2 min. In both cases, target and suspect screening, the *m*/*z* fragments presented in Tables S5 and S6 were compared with the experimental *m*/*z* fragments in the MS/MS spectrums of samples.

### 2.6. Statistical Analysis

ANOVA was applied using the Data Analysis tool of Microsoft Excel (Microsoft, Redmond, WA, USA). One-way ANOVA was performed to identify statistically significant differences, comparing the results at a 95% confidence level.

## 3. Results and Discussion

### 3.1. Target and Suspect Screening Results

From the initial target list of 14 compounds (Table S5), five biophenols were determined in the EVOOs: hydroxytyrosol and tyrosol from the group of phenolic alcohols, and luteolin and apigenin from the group of flavonoids and the lignan pinoresinol. The target screening results are summarized in Table 1. 

Fourteen suspect compounds were determined in the EVOOs. From the initial suspect list (Table S6), only the secoiridoid oleoside was not detected. The suspect screening results are summarized in Table 2. 

### 3.2. Quantification and Semiquantification Results

Standard calibration curves were prepared for the quantification or semiquantification of the biophenols detected in the EVOOs using commercial standards. Mixed standard working solutions of 0.1, 1.0, 2.5, 5, 8, 10, and 12 mg/L were prepared every day. Indicative normalized standard calibration curves (on the basis of 1.3 mg/L of syringaldehyde) constructed for tyrosol, hydroxytyrosol, oleuropein, apigenin, luteolin, and pinoresinol for one laboratory day are presented in Table S7.

Based on the results of target screening (Table 1), standard calibration curves of apigenin, luteolin, pinoresinol, tyrosol, and hydroxytyrosol were used to calculate the concentrations in the EVOOs. Regarding the suspect compounds, due to the absence of commercially available standards, the lignans syringaresinol, 1-hydroxypinoresinol, and 1-acetoxypinoresinol were quantified with the calibration curve of pinoresinol; 10-hydroxy decarboxymethyl oleuropein aglycone, oleocanthal, oleacein, hydroxytyrosol acetate, elenolic acid, and its hydroxylated form were quantified with the calibration curve of hydroxytyrosol; ligstroside aglycone, oleuropein aglycone, 10-hydoxy-10-methyl oleuropein aglycone, methyl oleuropein aglycone, and 10-hydroxy oleuropein aglycone were quantified using the calibration curve of oleuropein.

The quantified and semiquantified analytes are summarized in Table 3. The secoiridoids oleuropein aglycone and ligstroside aglycone were the main biophenols detected in the EVOOs. The average concentrations of oleuropein aglycone and ligstroside aglycone were 254 mg/kg and 127 mg/kg, respectively, followed by oleacein (20.1 mg/kg) and oleocanthal (13.9 mg/kg). Concerning the class of phenolic alcohols, the results showed that the average concentrations of tyrosol and hydroxytyrosol fluctuated at similar levels (2.53 mg/kg and 2.16 mg/kg, respectively). The flavonoids, apigenin and luteolin, were determined in all samples; however, the average concentration of apigenin (2.64 mg/kg) was higher than that of luteolin (1.87 mg/kg). From the class of lignans, 1-acetoxypinoresinol had a high concentration on average (21.5 mg/kg) in the samples. All compounds, apart from three, were detected in 90–100% of the samples. Syringaresinol and 1-hydroxypinoresinol were detected in about 80% of the samples. In addition, the hydroxylated form of elenolic acid was detected in a few EVOOs (<40% of the samples) and its concentration was low (≤1.21 mg/kg).

As shown in Table 3, from the sum of each individual compound that was determined, the bioactive content of samples was 475 mg/kg, on average, denoting their high quality and significant nutritional value.

Various analytical methods have been developed for the determination of phenolic content and/or individual compounds, making the comparison of results complicated because they are calculated in different ways. Table 4 presents the results of recent works reported in the literature regarding the bioactive content and concentrations of the compounds tyrosol, hydroxytyrosol, apigenin, luteolin, and pinoresinol determined in EVOOs/VOOs originating from Spain, Italy, Greece, Tunisia, Morocco, and Turkey. The advantage of this work compared to the literature is that the application of the UHPLC-QTOF-MS methodology enables the identification of analytes for which there are no available commercial standards, and therefore, they cannot be determined by traditional chromatographic methodologies. Consequently, HRMS is a powerful technique that leads to a thorough phenolic characterization of EVOOs.

### 3.3. Phenolic Content

- Comparison between the islands

**Figure 2:**
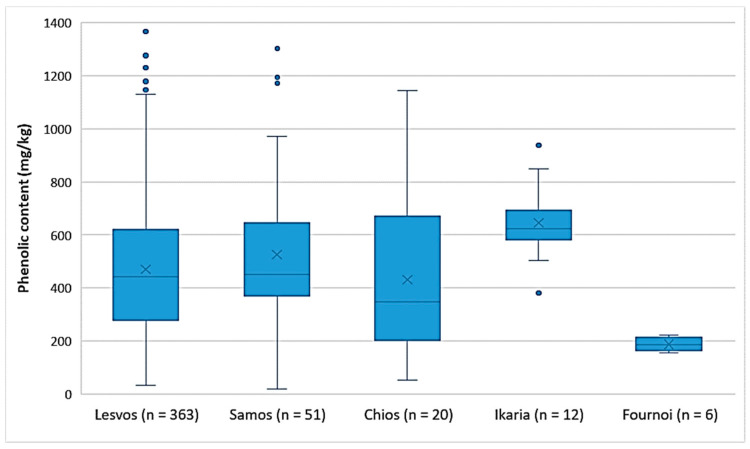
Comparison of the phenolic content (mg/kg) of EVOOs between the islands (Lesvos, Samos, Chios, Ikaria, and Fournoi) of the North Aegean Region.

As shown in Figure 2, the EVOOs from the islands of Lesvos, Samos, and Chios had the same variances in the phenolic content. This was also verified by ANOVA (*p* = 0.25). The EVOOs originating from Ikaria and Samos presented the highest phenolic content on average (646 mg/kg and 526 mg/kg, respectively), followed by the EVOOs from Lesvos (470 mg/kg) and Chios (431 mg/kg). In the case of Ikaria and Fournoi, no reliable comparison could be made using statistical analysis, because of the small number of samples (*n* ≤ 12) compared to the other islands. However, according to Figure 2 and Table 5, both Ikaria and Fournoi were within the range of the other three islands; the samples from Ikaria exhibited higher levels of phenolic constituents (380–939 mg/kg) and the samples from Fournoi had lower levels (155–222 mg/kg). Table 5 presents the statistical parameters median, mean, standard deviation (SD), and range of the phenolic content for each island.

- Comparison between the zones of Lesvos

**Figure 3:**
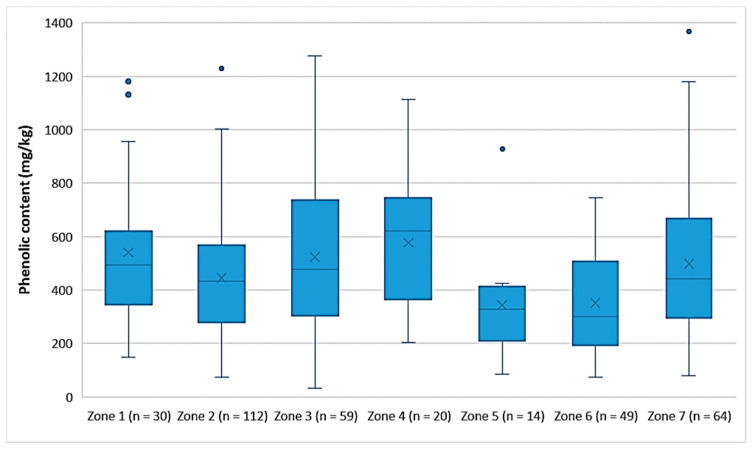
Comparison of the phenolic content (mg/kg) of EVOOs between the zones (Zones 1–7) of the island of Lesvos.

Figure 3 shows that the EVOOs from Zone 5 and Zone 6, which are located in the northern part of the island of Lesvos (Figure 1), had a lower phenolic content on average (343 mg/kg and 352 mg/kg, respectively) than did the EVOOs originating from the other geographical zones of the island. In the southern part of Lesvos (Zone 1–4, 7), the average phenolic content in the EVOOs ranged from 446 mg/kg (Zone 2) to 576 mg/kg (Zone 4). Τhe EVOOs from Zone 6 had a statistically significant difference compared to all other zones (*p* < 0.05), except for Zone 5 (*p* = 0.93). Moreover, the results of the statistical analysis demonstrated that the EVOOs from Zone 5 presented a statistically significant difference compared to the EVOOs from Zones 1, 3, and 4 (*p* < 0.05). The statistical parameters median, mean, SD, and range of the phenolic content for each zone are presented in Table 6.

### 3.4. Secoiridoids

In the EVOOs of this work, the most abundant group of biophenols was secoiridoids, followed by lignans, phenolic alcohols, and flavonoids. The percentage (%) of each group in the phenolic fraction is shown in Figure S1. This trend was observed both between the islands of the North Aegean Region and the zones of Lesvos. The island of Fournoi and Zone 5 of Lesvos were exceptions, as the percentage of phenolic alcohols was higher than the percentage of lignans. However, secoiridoids constituted the prevalent category of biophenols in all EVOOs, as their percentage was above 85% in all cases.

The major secoiridoids found in the EVOOs of this study were oleuropein aglycone, ligstroside aglycone, oleacein, and oleocanthal, as shown in Table 3. Based on the literature, the most abundant biophenols in VOOs are aglycones derived from the secoiridoids of drupes. These substances, such as oleuropein aglycone and ligstroside aglycone [66], have a significant role in the stability of VOO [10]. The decarboxymethylated dialdehyde form of oleuropein aglycone, oleacein, and the decarboxymethylated dialdehyde form of ligstroside aglycone, oleocanthal, are also very important compounds. Oleocanthal is responsible for the bitterness of VOO and also presents a high anti-inflammatory activity [67]. Oleacein also presents antioxidant properties [1].

#### 3.4.1. Major Secoiridoids—Differences between the Islands

- Oleuropein aglycone

**Figure 4:**
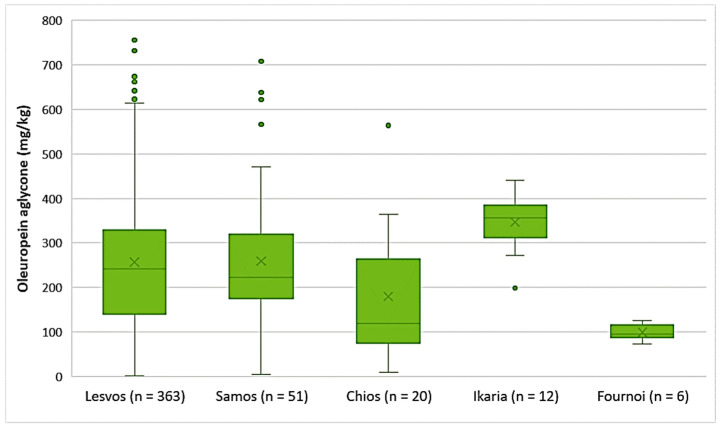
Concentration of oleuropein aglycone (mg/kg) in EVOOs from the islands of Lesvos, Samos, Chios, Ikaria, and Fournoi.

As shown in Figure 4, the highest average concentration of oleuropein aglycone was found in the EVOOs originating from Ikaria (347 mg/kg). On the other hand, there were EVOOs from the islands of Lesvos and Samos that presented higher concentrations of oleuropein aglycone with maximums of 756 mg/kg and 708 mg/kg, respectively. The average concentration of oleuropein aglycone in the EVOOs from Lesvos (256 mg/kg) and Samos (259 mg/kg) did not differ statistically (*p* = 0.91). However, the EVOOs from Chios, whose average concentration of oleuropein aglycone was 180 mg/kg, had a statistically significant difference compared to the EVOOs originating from Lesvos and Samos (*p* < 0.05). The EVOOs from Fournoi had the lowest average concentration of this secoiridoid (99.2 mg/kg).

- Ligstroside aglycone

**Figure 5:**
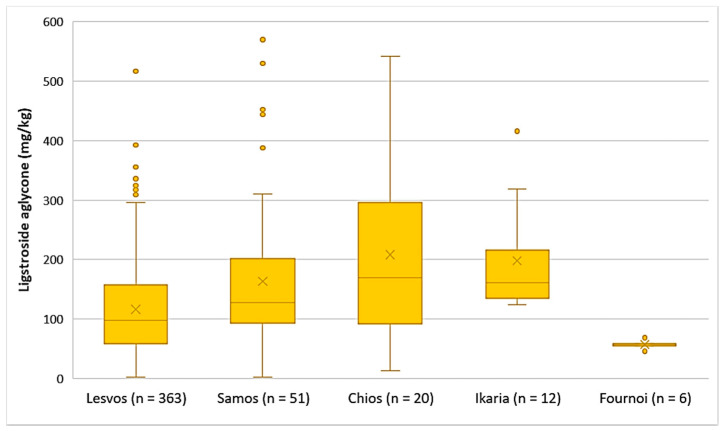
Concentration of ligstroside aglycone (mg/kg) in EVOOs from the islands of Lesvos, Samos, Chios, Ikaria, and Fournoi.

The EVOOs originating from Chios and Ikaria presented the highest average concentration of ligstroside aglycone (208 mg/kg and 198 mg/kg, respectively), followed by Samos (164 mg/kg) and Lesvos (116 mg/kg). Ligstroside aglycone was detected in low concentrations in the EVOOs from Fournoi, as its concentration ranged between 45.7 and 68.4 mg/kg (Figure 5). Moreover, the results of statistical analysis showed no significant difference between the EVOOs from Samos and Chios (*p* = 0.23). However, the samples originating from Lesvos presented a statistically significant difference compared to the samples from Samos and Chios (*p* < 0.05).

- Decarboxymethyl oleuropein aglycone (Oleacein)

**Figure 6:**
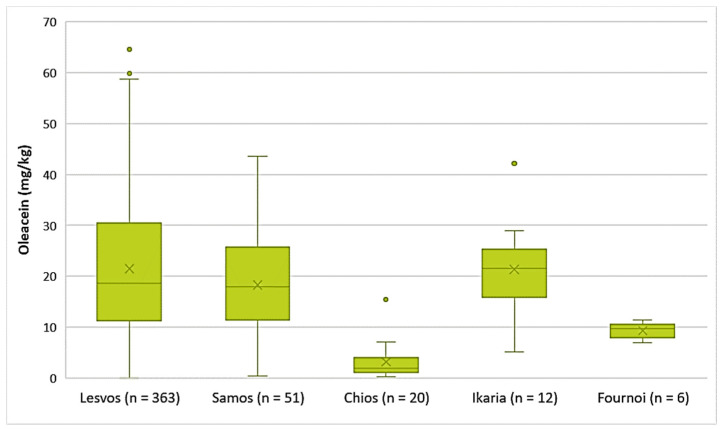
Concentration of oleacein (mg/kg) in EVOOs from the islands of Lesvos, Samos, Chios, Ikaria, and Fournoi.

The EVOOs from the islands of Lesvos, Ikaria, and Samos exhibited high concentrations of oleacein on average (21.5 mg/kg, 21.4 mg/kg, and 18.3 mg/kg, respectively). The highest concentration of oleacein was recorded in EVOO from Lesvos (64.6 mg/kg), as shown in Figure 6. Furthermore, the average concentration of oleacein was 9.33 mg/kg in the EVOOs from Fournoi and 3.15 mg/kg in the EVOOs from Chios. The differences between the EVOOs from Lesvos and Samos were not statistically significant (*p* = 0.11). On the contrary, the EVOOs from Chios presented a statistically significant difference compared to the EVOOs from Lesvos and Samos (*p* < 0.05).

- Decarboxymethyl ligstroside aglycone (Oleocanthal)

**Figure 7:**
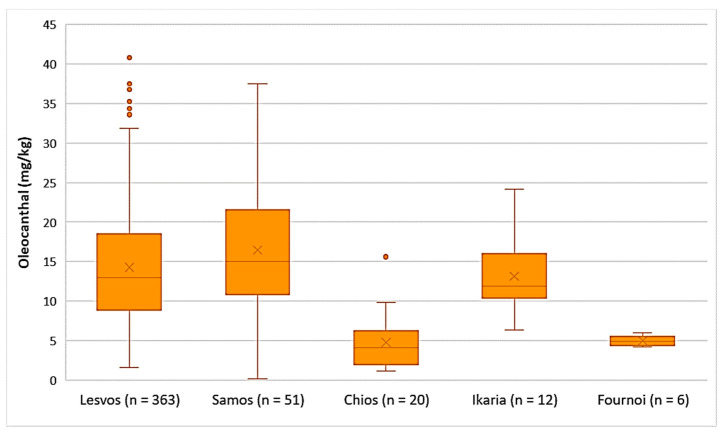
Concentration of oleocanthal (mg/kg) in EVOOs from the islands of Lesvos, Samos, Chios, Ikaria, and Fournoi.

As presented in Figure 7, the average concentration of oleocanthal was higher in the EVOOs from Samos (16.5 mg/kg), Lesvos (14.3 mg/kg), and Ikaria (13.2 mg/kg). The EVOOs originating from Chios and Fournoi had lower concentrations of oleocanthal on average (4.75 mg/kg and 4.99 mg/kg, respectively); however, the concentration levels ranged between 1.12 and 15.6 mg/kg in the case of Chios and between 4.20 and 6.01 mg/kg for the island of Fournoi. The differences in the concentration of oleocanthal were not statistically significant between the EVOOs from Lesvos and Samos (*p* = 0.053). On the contrary, the EVOOs originating from Chios presented a statistically significant difference compared to the EVOOs from Lesvos and Samos (*p* < 0.05).

#### 3.4.2. Major Secoiridoids—Differences between the Zones of Lesvos

- Oleuropein aglycone

**Figure 8:**
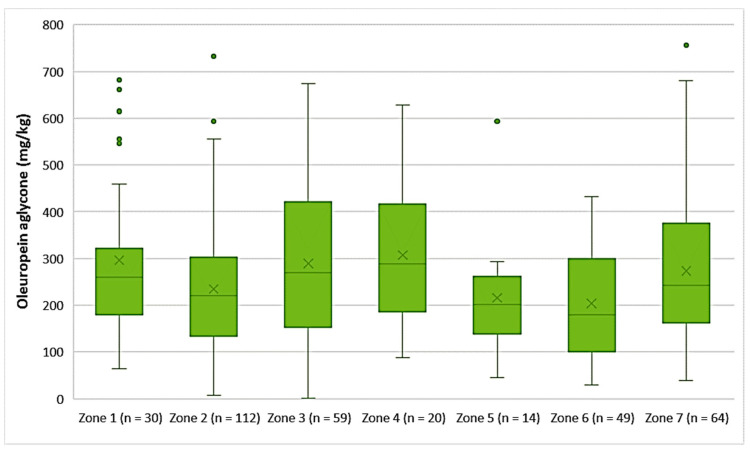
Concentration of oleuropein aglycone (mg/kg) in EVOOs from the seven zones (Zones 1–7) of the island of Lesvos.

The EVOOs from Zones 1, 3, 4, and 7 exhibited high concentrations of oleuropein aglycone on average (296 mg/kg, 290 mg/kg, 308 mg/kg, and 274 mg/kg, respectively). Moreover, Figure 8 shows that the average concentration of oleuropein aglycone was 235 mg/kg in the EVOOs from Zone 2, 216 mg/kg in the EVOOs from Zone 5, and 204 mg/kg in the EVOOs from Zone 6. The highest concentration of this secoiridoid was found in EVOOs originating from Zone 7 (756 mg/kg) and Zone 2 (732 mg/kg), while the lowest concentration was recorded in EVOOs from Zone 3 (1.44 mg/kg) and Zone 2 (7.87 mg/kg). No statistically significant difference was observed between the samples from Zones 1, 3, 4, 5, and 7 (*p* > 0.05). In addition, the EVOOs from Zones 2, 5, and 6 had no statistically significant difference (*p* > 0.05).

- Ligstroside aglycone

**Figure 9:**
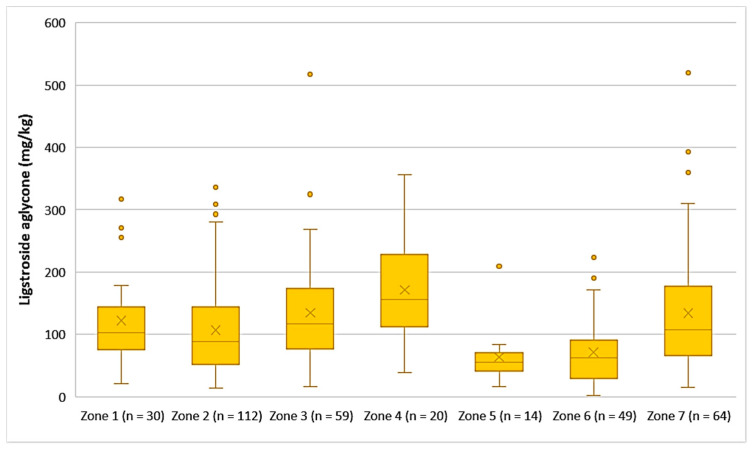
Concentration of ligstroside aglycone (mg/kg) in EVOOs from the seven zones (Zones 1–7) of the island of Lesvos.

The average concentration of ligstroside aglycone was higher in the EVOOs from Zone 4 (171 mg/kg), followed by Zone 3 (135 mg/kg), Zone 7 (134 mg/kg), Zone 1 (123 mg/kg), and Zone 2 (107 mg/kg). Ligstroside aglycone was detected in low concentrations in the EVOOs from Zone 5 (63.9 mg/kg) and Zone 6 (71.2 mg/kg). Moreover, according to Figure 9, the highest concentrations of ligstroside aglycone were found in EVOOs originating from Zone 3 (517 mg/kg) and Zone 7 (520 mg/kg), while the lowest concentration was recorded in EVOO originating from Zone 6 (2.33 mg/kg). Statistical analysis showed no significant difference between the samples from Zones 1, 3, 4, and 7 (*p* > 0.05). The EVOOs originating from Zone 2 presented a statistically significant difference compared to the EVOOs from the other zones, apart from Zone 1 (*p* = 0.31). Furthermore, the samples from Zone 5 and Zone 6 did not differ statistically (*p* = 0.63).

- Decarboxymethyl oleuropein aglycone (Oleacein)

**Figure 10:**
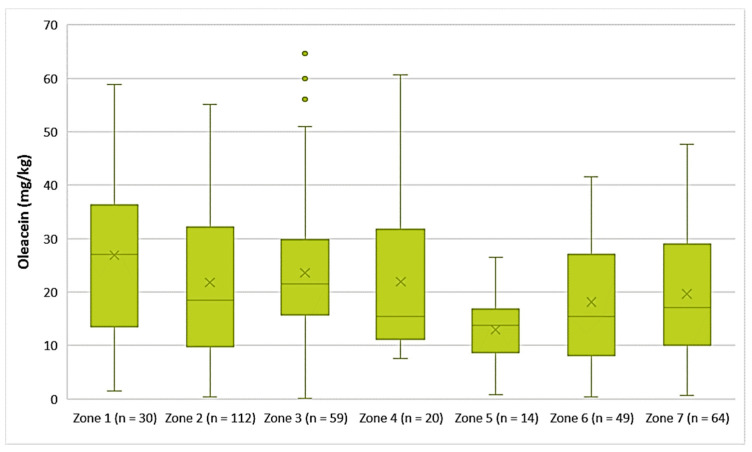
Concentration of oleacein (mg/kg) in EVOOs from the seven zones (Zones 1–7) of the island of Lesvos.

The EVOOs from Zone 1 exhibited the highest average concentration of oleacein (26.9 mg/kg) and the EVOOs from Zone 5 had the lowest average concentration of oleacein (13.0 mg/kg). As for the EVOOs from the other zones, the average concentration of oleacein ranged from 18.1 mg/kg (Zone 6) to 23.6 mg/kg (Zone 3). As shown in Figure 10, the highest concentrations of oleacein were noted in EVOO from Zone 3 (64.6 mg/kg) and Zone 4 (60.7 mg/kg). The differences between the samples from Zones 1, 2, 3, 4, and 7 were not statistically significant (*p* > 0.05). The result from ANOVA was the same for the EVOOs from Zones 5, 6, and 7.

- Decarboxymethyl ligstroside aglycone (Oleocanthal)

**Figure 11:**
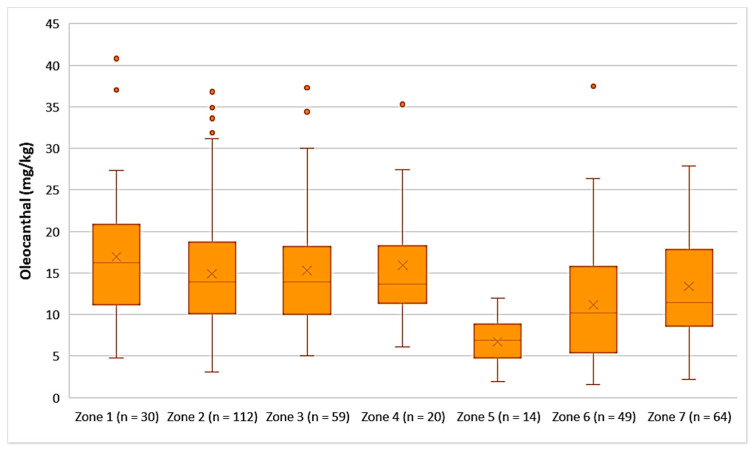
Concentration of oleocanthal (mg/kg) in EVOOs from the seven zones (Zones 1–7) of the island of Lesvos.

As shown in Figure 11, the highest average concentration of oleocanthal was found in the EVOOs originating from Zone 1 (16.9 mg/kg). Oleocanthal was detected in low concentrations in the EVOOs from Zone 5, as the average concentration of this secoiridoid was 6.71 mg/kg and the maximum value did not exceed 12.0 mg/kg. ANOVA showed that the EVOOs from Zone 5 presented a statistically significant difference compared to the EVOOs from the other zones (*p* < 0.05). The EVOOs from Zone 6, which had the second lowest average concentration of oleacein (11.2 mg/kg), did not differ statistically only with the EVOOs from Zone 7 (*p* = 0.11). On the other hand, the samples from Zones 1, 2, 3, 4, and 7 presented no statistically significant difference between them (*p* > 0.05).

### 3.5. Phenolic Profile

**Figure 12:**
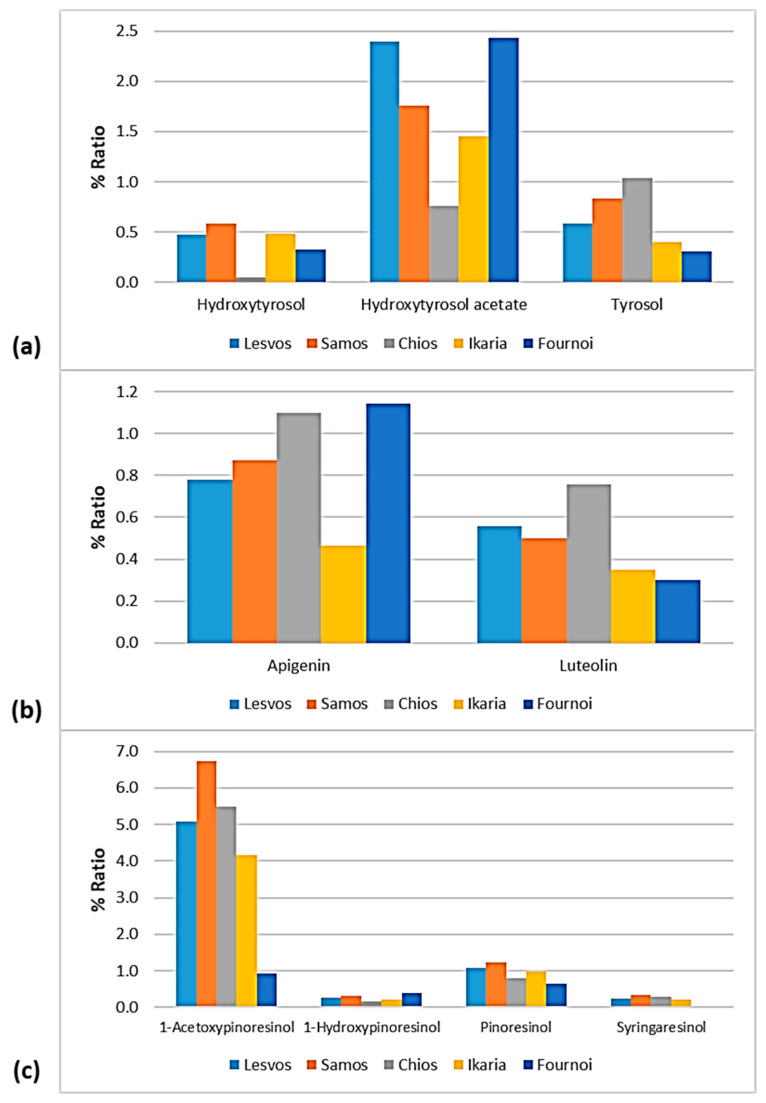
% Ratio of the compounds from the class of (**a**) phenolic alcohols (hydroxytyrosol, hydroxytyrosol acetate, and tyrosol), (**b**) flavonoids (apigenin and luteolin), and (**c**) lignans (1-acetoxypinoresinol, 1-hydroxypinoresinol, pinoresinol, and syringaresinol) to the phenolic content in the EVOOs between the islands (Lesvos, Samos, Chios, Ikaria, and Fournoi) of the North Aegean Region.

**Figure 13:**
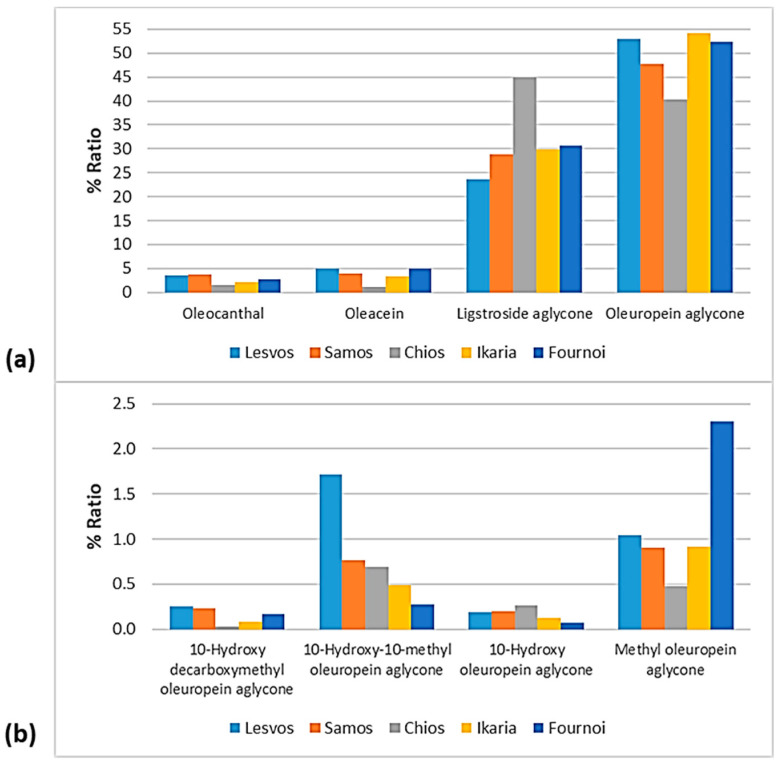
% Ratio of the secoiridoids (**a**) oleocanthal, oleacein, ligstroside aglycone, and oleuropein aglycone; and (**b**) methyl oleuropein aglycone, 10-hydroxy-10-methyl oleuropein aglycone, 10-hydroxy decarboxymethyl oleuropein aglycone, and 10-hydroxy oleuropein aglycone to the phenolic content in the EVOOs between the islands (Lesvos, Samos, Chios, Ikaria, and Fournoi) of the North Aegean Region.

**Figure 14:**
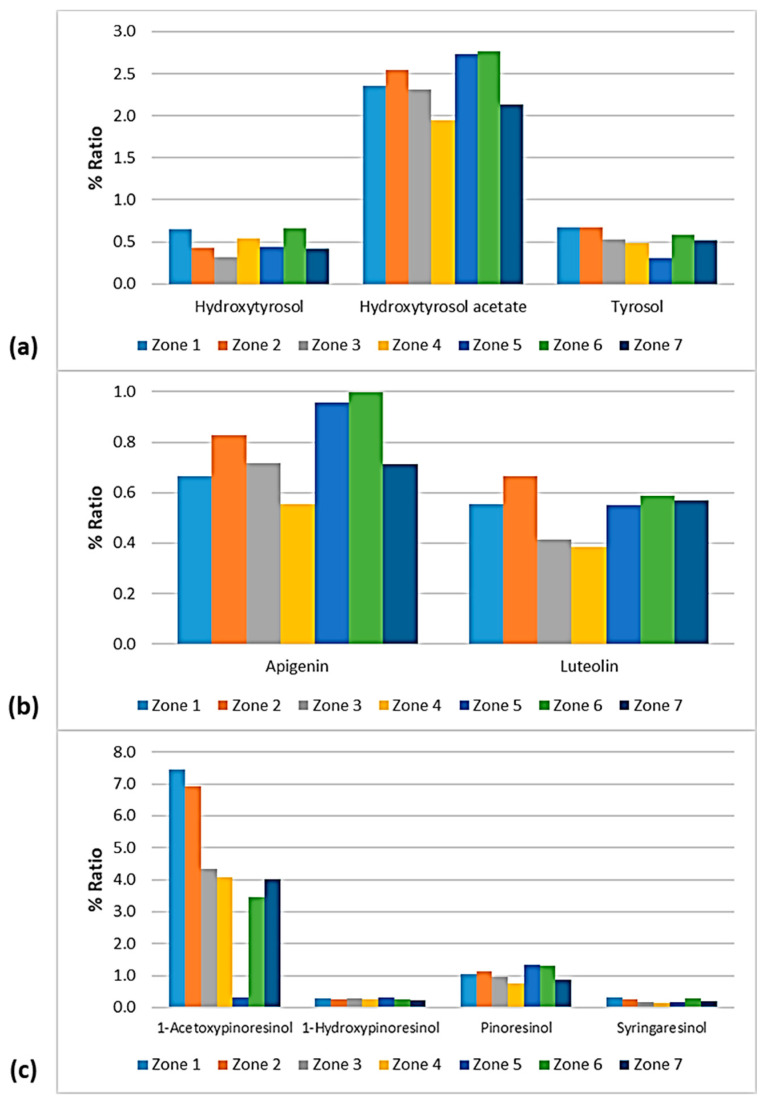
% Ratio of the compounds from the class of (**a**) phenolic alcohols (hydroxytyrosol, hydroxytyrosol acetate, and tyrosol); (**b**) flavonoids (apigenin and luteolin); and (**c**) lignans (1-acetoxypinoresinol, 1-hydroxypinoresinol, pinoresinol, and syringaresinol) to the phenolic content in the EVOOs between the zones of Lesvos (Zones 1–7).

**Figure 15:**
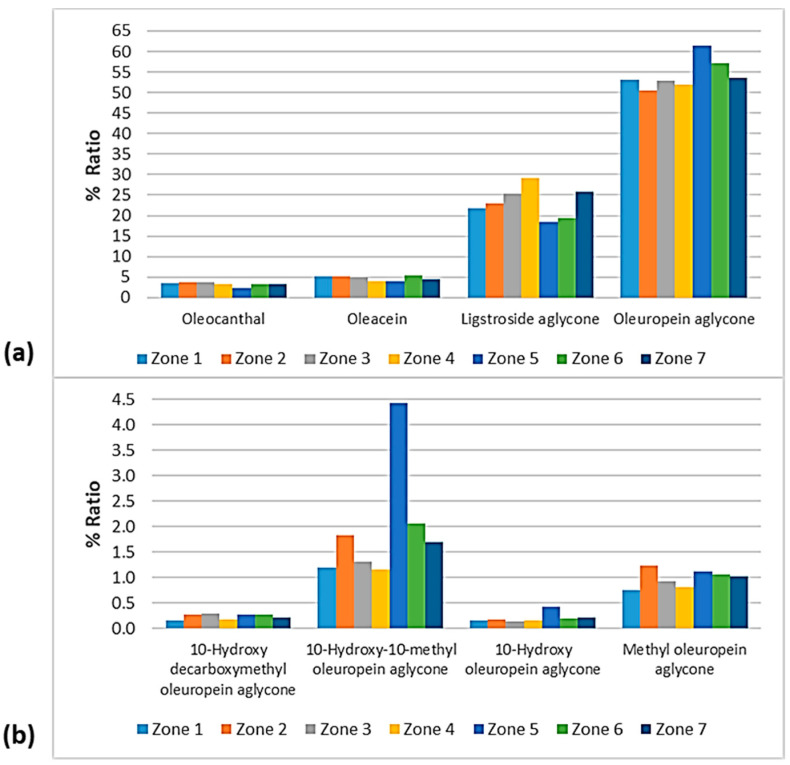
% Ratio of the secoiridoids (**a**) oleocanthal, oleacein, ligstroside aglycone, and oleuropein aglycone; and (**b**) methyl oleuropein aglycone, 10-hydroxy-10-methyl oleuropein aglycone, 10-hydroxy decarboxymethyl oleuropein aglycone, and 10-hydroxy oleuropein aglycone to the phenolic content in the EVOOs between the zones of Lesvos (Zones 1–7).

The phenolic profile of VOOs/EVOOs constitutes a significant factor, as differences in the distribution of the biophenols can lead to discriminations between samples. For this reason, the present work was extended to the study of each compound individually. The figures of this section (Figure 12, Figure 13, Figure 14 and Figure 15) present the % ratio of each compound detected in the EVOOs to the overall phenolic content, from the four main categories of biophenols (secoiridoids, phenolic alcohols, lignans, and flavonoids). It was considered appropriate to study the % percentage of compounds, not the concentration in mg/kg, to compare the contribution of each compound to the phenolic content of EVOOs.

#### 3.5.1. Phenolic Profile—Comparison between the Islands

As for the class of phenolic alcohols, as shown in Figure 12, hydroxytyrosol ranged between 0.32 and 0.59% in the EVOOs between the islands, apart from the samples from Chios whose percentage of hydroxytyrosol was very low (0.05%). Hydroxytyrosol acetate was also low in the EVOOs from Chios (0.76%). On the other hand, the EVOOs originating from Lesvos and Fournoi exhibited high levels of hydroxytyrosol acetate (2.40% and 2.43%, respectively), followed by the EVOOs from Samos (1.75%) and Ikaria (1.45%). Moreover, tyrosol was lower in the EVOOs from Ikaria and Fournoi (<0.5%) and higher in the EVOOs from Chios (1.04%).

Both flavonoids, luteolin and luteolin, were higher in the EVOOs from Chios (1.10% and 0.76%, respectively). Apigenin was also high in the EVOOs from Fournoi (1.14%). The EVOOs originating from Ikaria presented a low ratio of flavonoids (0.46% for apigenin and 0.35% for luteolin). As for the EVOOs from Lesvos and Samos, they were found in the middle of the scale for both flavonoids. In all the islands, the percentage of apigenin was higher than that of luteolin.

The differences were slight between the islands about the lignans 1-hydroxypinoresinol and syringaresinol. However, as presented in Figure 12, syringaresinol was not detected in the EVOOs originating from Fournoi. Furthermore, 1-acetoxypinoresinol and pinoresinol were higher in the EVOOs from Samos (6.74% and 1.22%, respectively) and lower in the EVOOs from Fournoi (0.91% and 0.66%, respectively).

As for the secoiridoids, according to Figure 13, oleuropein aglycone did not show significant differences between the islands, as it ranged between 47.8 and 54.1%, with the only exception of the EVOOs originating from Chios (40.3%). On the contrary, the EVOOs from Chios presented the highest percentage of ligstroside aglycone (45.0%), whereas the percentage ranged between 23.7 and 30.7% for the other islands. Oleacein and oleocanthal were lower in the EVOOs originating from Chios (1.06% and 1.59%, respectively). The former was higher in the EVOOs from Lesvos (4.94%) and Fournoi (4.96%), and the latter was higher in the EVOOs from Samos (3.81%) and Lesvos (3.49%). Moreover, 10-hydorxy-10-methyl oleuropein aglycone was higher in the EVOOs from Lesvos (1.72%) and lower in the EVOOs from Fournoi (0.28%). Methyl oleuropein aglycone was higher in the EVOOs from Fournoi (2.31%) and lower in the EVOOs from Chios (0.48%). In the case of 10-hydroxy oleuropein aglycone and 10-hydroxy decarboxymethyl oleuropein aglycone, the fluctuations were slight between the islands (≤0.25%).

#### 3.5.2. Phenolic Profile—Comparison between the Zones of Lesvos

As presented in Figure 14, the phenolic alcohols hydroxytyrosol and tyrosol fluctuated at the same levels (0.30–0.67%) between the seven geographical areas of Lesvos. The former showed a lower percentage in the EVOOs from Zone 3 and higher percentages in the EVOOs from Zone 1 and Zone 6, and the latter exhibited a lower ratio in the EVOOs from Zone 5 and a higher ratio in the EVOOs from Zone 1 and Zone 2. Moreover, hydroxytyrosol acetate was lower in the samples from Zone 4 (1.95%) and higher in the samples from Zone 5 (2.74%) and Zone 6 (2.77%).

The EVOOs from Zone 4 presented the lowest percentages for both flavonoids; apigenin was 0.56% and luteolin was 0.38%. Luteolin was also low in the case of EVOOs from Zone 3 (0.41%). On the other hand, apigenin was higher in the EVOOs from Zone 5 (0.96%) and Zone 6 (1.00%) and luteolin was higher in the EVOOs originating from Zone 2 (0.67%).

The differences between the seven zones of Lesvos were not significant for the lignans 1-hydroxypinoresinol and syringaresinol, as shown in Figure 14. 1-Acetoxypinoresinol was higher in the EVOOs from Zone 1 (7.44%) and Zone 2 (6.93%), and very low in the EVOOs from Zone 5 (0.33%). Furthermore, pinoresinol was lower in the EVOOs from Zone 4 and Zone 7 (0.76% and 0.88%, respectively), and higher in the EVOOs from Zone 5 and Zone 6 (1.33% and 1.30%, respectively). 

Figure 15 shows that oleuropein aglycone was higher in the EVOOs from Zone 5 and Zone 6 (61.4% and 57.1%, respectively). In addition, the EVOOs from those geographical areas had the lowest percentages of ligstroside aglycone (18.6% and 19.3%, respectively). The highest percentage of ligstroside aglycone occurred in the EVOOs originating from Zone 4 (29.2%). Moreover, oleocanthal and oleacein had the lowest percentages in the EVOOs from Zone 5 (2.30% and 3.93%, respectively). The secoiridoid oleacein was also low in the EVOOs from Zone 4 (3.97%) and Zone 7 (4.38%). As for the other secoiridoids, specifically, methyl oleuropein aglycone, 10-hydorxy-10-methyl oleuropein aglycone, 10-hydroxy decarboxymethyl oleuropein aglycone, and 10-hydroxy oleuropein aglycone, the differences were slight between the seven zones. Τhe only exception was Zone 5, where the samples exhibited an impressively high percentage of 10-hydorxy-10-methyl oleuropein aglycone (4.44%) compared to the EVOOs from the other geographical zones of the island of Lesvos.

## 4. Conclusions

Nineteen biophenols were determined in the analyzed EVOOs originating from the islands of the North Aegean Region in Greece (Lesvos, Samos, Chios, Ikaria, and Fournoi). The sum of the determined biophenols was calculated to establish a trend between the EVOOs and the geographical origin. The phenolic content of the EVOOs originating from Lesvos ranged between 32 and 1368 mg/kg, between 20 and 1304 mg/kg for those originating from Samos, and between 52 and 1146 mg/kg for EVOOs originating from Chios. The EVOOs originating from Ikaria also exhibited a high phenolic content as the sum of the individual quantified and semiquantified biophenols ranged from 380 to 939 mg/kg, while the EVOOs originating from Fournoi presented a lower bioactive content (155–222 mg/kg).

Concerning the four major secoiridoids, the samples from Lesvos, Samos, and Ikaria exhibited higher average concentrations of oleacein and oleocanthal, with EVOOs from Lesvos recording the highest values. Furthermore, the phenolic profile of EVOOs presented differences between the five islands. The EVOOs from Chios exhibited the lowest percentage of the phenolic alcohols hydroxytyrosol and hydroxytyrosol acetate, and the highest tyrosol percentage. The samples from Ikaria presented low percentages of flavonoids. Moreover, the EVOOs from Lesvos exhibited a high percentage of 10-hydroxy-10-methyl oleuropein aglycone, while the EVOOs from Fournoi presented a high percentage of methyl oleuropein aglycone and a low percentage of the lignan 1-acetoxypinoresinol. The latter recorded the highest percentage in the case of EVOOs from Samos.

As for the zones of Lesvos, the results showed that the EVOOs from the southern part of the island had a higher bioactive content than the EVOOs from the northern part (Zone 5 and Zone 6). Furthermore, the EVOOs from Zone 5 and Zone 6 exhibited the highest percentages of oleuropein aglycone and the lowest percentages of ligstroside aglycone, compared to the other geographical zones. On the other hand, the EVOOs from Zone 4 presented low percentages of the flavonoids luteolin and apigenin, whereas the percentage of 1-acetoxypinoresinol was high in the EVOOs from Zone 1 and Zone 2, and very low in the EVOOs from Zone 5. 

In conclusion, the observed differences in the phenolic content and profile can lead to discriminations of EVOOs both between the islands of North Aegean and between the different locations of Lesvos. Therefore, taking into account the results of the present study, it is strongly supported that the geographical origin affects the levels of biophenols of EVOOs.

## Figures and Tables

**Table 1:** Results of target screening for EVOOs.

Analyte	Molecular Formula	*m*/*z* [M–H]^−^ Theoretical	*m*/*z* [M–H]^−^ Experimental	t_R_ Standard (min)	Δt_R_ (min)
**Phenolic alcohols**					
Hydroxytyrosol	C_8_H_10_O_3_	153.0557	153.0555	3.53	−0.01
Tyrosol	C_8_H_10_O_2_	137.0608	137.0607	4.07	+0.02
**Flavonoids**					
Apigenin	C_15_H_10_O_5_	269.0455	269.0455	8.24	−0.03
Luteolin	C_15_H_10_O_6_	285.0404	285.0405	7.55	−0.04
**Lignans**					
Pinoresinol	C_20_H_22_O_6_	357.1343	357.1341	6.49	+0.01

**Table 2:** Results of suspect screening for EVOOs.

Analyte	Molecular Formula	*m*/*z* [M–H]^−^ Calculated	*m*/*z* [M–H]^−^ Experimental	t_R_ (min)	Δt_R_ (min)
**Phenolic alcohols**					
Hydroxytyrosol acetate	C_10_H_12_O_4_	195.0663	195.0663	6.71	+0.07
**Secoiridoids**					
Decarboxymethyl ligstroside aglycone (Oleocanthal)	C_17_H_20_O_5_	303.1237	303.1236	6.42	+0.02
Decarboxymethyl oleuropein aglycone (Oleacein)	C_17_H_20_O_6_	319.1187	319.1185	5.61	+0.04
10-Hydroxy-10-methyl oleuropein aglycone	C_20_H_24_O_9_	407.1347	407.1347	6.71	+0.04
10-Hydroxy decarboxymethyl oleuropein aglycone	C_17_H_20_O_7_	335.1136	335.1135	4.28	+0.16
10-Hydroxy oleuropein aglycone	C_19_H_22_O_9_	393.1191	393.1190	4.82	−0.12
Ligstroside aglycone	C_19_H_22_O_7_	361.1292	361.1291	6.63	+0.06
Methyl oleuropein Aglycone	C_20_H_24_O_8_	391.1398	391.1396	7.51	+0.12
Oleuropein aglycone	C_19_H_22_O_8_	377.1241	377.1240	7.29	+0.07
**Lignans**					
1-Acetoxypinoresinol	C_22_H_24_O_8_	415.1398	415.1397	6.42	+0.08
1-Hydroxypinoresinol	C_20_H_22_O_7_	373.1292	373.1290	6.39	+0.02
Syringaresinol	C_22_H_26_O_8_	417.1554	417.1557	6.18	+0.01
**Other compounds**					
Elenolic acid	C_11_H_14_O_6_	241.0717	241.0716	4.51	−0.05
Hydroxylated form of elenolic acid	C_11_H_14_O_7_	257.0667	257.0663	1.36	+0.01

**Table 3:** Quantification and semiquantification results in EVOOs.

Compound	Average Concentration (mg/kg)	Concentration Range (mg/kg)	Number of Samples ^2^
**Phenolic alcohols**			
Hydroxytyrosol	2.16	ND ^1^–27.4	441
Hydroxytyrosol acetate	9.61	0.06–34.5	452
Tyrosol	2.53	0.17–18.8	452
**Secoiridoids**			
10-Hydroxy-10-methyl oleuropein aglycone	6.58	ND ^1^–92.2	447
10-Hydroxy decarboxymethyl oleuropein aglycone	1.05	ND ^1^–7.08	442
10-Hydroxy oleuropein aglycone	0.93	ND ^1^–16.7	407
Ligstroside aglycone	127	2.33–570	452
Methyl oleuropein aglycone	4.56	ND ^1^–26.9	447
Oleacein	20.1	0.03–64.6	452
Oleocanthal	13.9	0.19–40.8	452
Oleuropein aglycone	254	1.44–756	452
**Flavonoids**			
Apigenin	2.64	1.97–5.10	452
Luteolin	1.87	0.13–7.71	452
**Lignans**			
1-Acetoxypinoresinol	21.5	ND ^1^–96.9	443
1-Hydroxypinoresinol	0.92	ND ^1^–6.65	353
Pinoresinol	4.19	0.25–15.0	452
Syringaresinol	0.99	ND ^1^–4.61	359
**Other compounds**			
Elenolic acid	0.88	ND ^1^–8.84	448
Hydroxylated form of elenolic acid	0.04	ND ^1^–1.21	168
**Phenolic content**	475	20–1368	452

^1^ ND: not detected; ^2^ number of EVOO samples in which each analyte was detected.

**Table 4:** Phenolic content and concentrations of tyrosol, hydroxytyrosol, apigenin, luteolin, and pinoresinol (mg/kg) in VOOs/EVOOs from Mediterranean countries.

Country	Analytical Method	Phenolic Content (mg/kg)	Tyrosol (mg/kg)	Hydroxytyrosol (mg/kg)	Apigenin (mg/kg)	Luteolin (mg/kg)	Pinoresinol (mg/kg)	Reference
Spain	UHPLC-MS/MS	62.8–181	ND ^1^–30.0	6.94–72.7	0.20–3.29	0.85–3.62	ND ^1^–7.01	[29]
Spain	UHPLC-MS/MS	138–186	1.10–1.57	2.6–4.1	0.75–0.82	1.9–2.3	2.94–4.10	[25]
Spain	UHPLC-MS/MS	289–567		0.46–1.27	0.49–2.23	0.32–0.69	5.57–8.24	[57]
Italy	HPLC-DAD	232–830	2.9–37.9	2.6–26.6	0.3–19.7	0.7–5.4		[58]
	NMR	247–851						
Italy	HPLC-DAD/MS	257–1220	1.6–42.9	0.9–29.5	ND ^1^–3.2	0.7–5.4		[31]
Italy	HPLC-DAD	814–5920	0.03–62.3	0.72–62.7	1.05–2.91	11.6–16.8		[32]
Italy	HPLC-DAD/MS		5.4–124	22.8–208	ND ^1^–19.5	1.4–53.0		[59]
Greece	NMR	≤4003 (mean: 483)						[60]
Tunisia	LC-MS	109–493	1.32–23.2	2.71–22.6	2.32–2.96	4.35–17.1	ND ^1^–6.49	[61]
Tunisia	LC-MS		1.77–3.12	3.84–7.18	0.09–1.50	0.74–6.54	ND ^1^–1.23	[24]
Tunisia	HPLC-DAD	136–218	16.1–40.5	9.61–24.1	0.05–1.02	1.06–5.93	2.66–23.8	[26]
Morocco	LC-MS/MS		3.50–19.6	5.51–18.3	0.09–0.43	0.93–3.51	0.14–0.76	[62]
Morocco	HPLC-DAD		12–29.4			1.02–4.1		[63]
Turkey	LC-MS/MS	42.2–95.9	6.93–14.9	0.78–17.4	1.00–8.32	1.54–10.2		[64]
Turkey	HPLC-DAD		1.70–10.7	3.16–7.36	0.77–2.64	0.82–1.66		[35]
Mediterranean area	HPLC-DAD	26–1410			0.10–11.4	0.44–20.1	ND ^1^–23.9	[65]

^1^ ND: not detected.

**Table 5:** Statistical parameters for the phenolic content of EVOOs for each island.

Island	Median (mg/kg)	Mean (mg/kg)	SD (mg/kg)	Range (mg/kg)
Lesvos (*n* = 363)	441	470	246	32–1368
Samos (*n* = 51)	452	526	275	20–1304
Chios (*n* = 20)	347	431	299	52–1146
Ikaria (*n* = 12)	623	646	149	380–939
Fournoi (*n* = 6)	186	188	29	155–222

**Table 6:** Statistical parameters for the phenolic content of EVOOs for the seven zones of Lesvos.

Zone	Median (mg/kg)	Mean (mg/kg)	SD (mg/kg)	Range (mg/kg)
Zone 1 (*n* = 30)	493	541	275	149–1181
Zone 2 (*n* = 112)	434	446	220	75–1230
Zone 3 (*n* = 59)	478	523	274	32–1277
Zone 4 (*n* = 20)	622	576	240	203–1115
Zone 5 (*n* = 14)	329	343	202	85–929
Zone 6 (*n* = 49)	302	352	195	74–746
Zone 7 (*n* = 64)	443	498	271	78–1368

## Supplementary Materials

The following are available online at https://www.mdpi.com/article/10.3390/foods10092102/s1, Figure S1: Types of phenolic compounds (%) in EVOOs between the (a) islands; (b) zones of Lesvos, Table S1: Gradient program of mobile phase, Table S2: The main olive tree varieties and the number of EVOO samples in the five islands of the North Aegean Region, Table S3: Number of samples in each geographical zone of the island of Lesvos, Table S4: QC results, Table S5: List of target compounds, Table S6: List of suspect compounds, Table S7: Standard calibration curve and coefficient of determination (r^2^) for tyrosol, hydroxytyrosol, oleuropein, apigenin, luteolin, and pinoresinol.

## References

[1] Miho H., Díez C.M., Mena-Bravo A., Sánchez de Medina V., Moral J., Melliou E., Magiatis P., Rallo L., Barranco D., Priego-Capote F. (2018). Cultivar influence on variability in olive oil phenolic profiles determined through an extensive germplasm survey. Food Chem..

[2] Sánchez De Medina V., Priego-Capote F., De Castro M.D.L. (2015). Characterization of monovarietal virgin olive oils by phenols profiling. Talanta.

[3] Franco M.N., Galeano-Díaz T., López Ó., Fernández-Bolaños J.G., Sánchez J., de Miguel C., Gil M.V., Martín-Vertedor D. (2014). Phenolic compounds and antioxidant capacity of virgin olive oil. Food Chem..

[4] Antonini E., Farina A., Leone A., Mazzara E., Urbani S., Selvaggini R., Servili M., Ninfali P. (2015). Phenolic compounds and quality parameters of family farming versus protected designation of origin (PDO) extra-virgin olive oils. J. Food Compos. Anal..

[5] Gomez-Rico A., Inarejos-Garcia A., Desamparados Salvador M., Fregapane G. (2009). Effect of malaxation conditions on phenol and volatile profiles in olive paste and the corresponding virgin olive oils (*Olea europaea* L. Cv. Cornicabra). J. Agric. Food Chem..

[6] Rios J.J., Gil M.J., Gutierrez-Rosales F. (2005). Solid-phase extraction gas chromatography-ion trap-mass spectrometry qualitative method for evaluation of phenolic compounds in virgin olive oil and structural confirmation of oleuropein and ligstroside aglycons and their oxidation products. J. Chromatogr. A.

[7] Guerfel M., Ouni Y., Taamalli A., Boujnah D., Stefanoudaki E., Zarrouk M. (2009). Effect of location on virgin olive oils of the two main Tunisian olive cultivars. Eur. J. Lipid Sci. Technol..

[8] Gutiérrez-Rosales F., Ríos J.J., Gómez-Rey M.L. (2003). Main polyphenols in the bitter taste of virgin olive oil. Structural confirmation by on-line high-performance liquid chromatography electrospray ionization mass spectrometry. J. Agric. Food Chem..

[9] Basdeki E., Salis C., Hagidimitriou M. (2020). The effects of Mediterranean diet and EVOO consumption in relation to human health. Not. Sci. Biol..

[10] Koseoglu O., Unal M.K. (2008). The effect of phenolic compounds on the quality and stability of virgin olive oil. Acta Hortic..

[11] Romani A., Ieri F., Urciuoli S., Noce A., Marrone G., Nediani C., Bernini R. (2019). Health effects of phenolic compounds found in extra-virgin olive oil, by-products, and leaf of *Olea europaea* L.. Nutrients.

[12] Hashmi M.A., Khan A., Hanif M., Farooq U., Perveen S. (2015). Traditional uses, phytochemistry, and pharmacology of *Olea europaea* (olive). Evid.-Based Complement. Altern. Med..

[13] Brenes M., Hidalgo F.J., García A., Rios J.J., García P., Zamora R., Garrido A. (2000). Pinoresinol and 1-acetoxypinoresinol, two new phenolic compounds identified in olive oil. J. Am. Oil Chem. Soc..

[14] Ocakoglu D., Tokatli F., Ozen B., Korel F. (2009). Distribution of simple phenols, phenolic acids and flavonoids in Turkish monovarietal extra virgin olive oils for two harvest years. Food Chem..

[15] Noce A., Marrone G., Urciuoli S., Di Daniele F., Di Lauro M., Zaitseva A.P., Di Daniele N., Romani A. (2021). Usefulness of extra virgin olive oil minor polar compounds in the management of chronic kidney disease patients. Nutrients.

[16] El Riachy M., Hamade A., Ayoub R., Dandachi F., Chalak L. (2019). Oil content, fatty acid and phenolic profiles of some olive varieties growing in Lebanon. Front. Nutr..

[17] Di Lecce G., Piochi M., Pacetti D., Frega N., Bartolucci E., Scortichini S., Fiorini D. (2020). Eleven monovarietal extra virgin olive oils from olives grown and processed under the same conditions: Effect of the cultivar on the chemical composition and sensory traits. Foods.

[18] Quintero-Flórez A., Pereira-Caro G., Sánchez-Quezada C., Moreno-Rojas J.M., Gaforio J.J., Jimenez A., Beltrán G. (2017). Effect of olive cultivar on bioaccessibility and antioxidant activity of phenolic fraction of virgin olive oil. Eur. J. Nutr..

[19] Artajo L.-S., Romero M.-P., Tovar M.-J., Motilva M.-J. (2006). Effect of irrigation applied to olive trees (*Olea europaea* L.) on phenolic compound transfer during olive oil extraction. Eur. J. Lipid Sci. Technol..

[20] Caruso G., Gucci R., Sifola I., Selvaggini R., Urbani S., Esposto S., Servili M. (2017). Irrigation and fruit canopy position modify oil quality of olive trees (cv. Frantoio). J. Sci. Food Agric..

[21] Caponio F., Gomes T., Pasqualone A. (2001). Phenolic compounds in virgin olive oils: Influence of the degree of olive ripeness on organoleptic characteristics and shelf-life. Eur. Food Res. Technol..

[22] Kula Ö., Yıldırım A., Yorulmaz A., Duran M., Mutlu İ., Kıvrak M. (2018). Effect of crushing temperature on virgin olive oil quality and composition. Grasas y Aceites.

[23] Cecchi L., Migliorini M., Zanoni B., Breschi C., Mulinacci N. (2018). An effective HPLC-based approach for the evaluation of the content of total phenolic compounds transferred from olives to virgin olive oil during the olive milling process. J. Sci. Food Agric..

[24] Ouni Y., Taamalli A., Gómez-Caravaca A.M., Segura-Carretero A., Fernández-Gutiérrez A., Zarrouk M. (2011). Characterisation and quantification of phenolic compounds of extra-virgin olive oils according to their geographical origin by a rapid and resolutive LC-ESI-TOF MS method. Food Chem..

[25] Bakhouche A., Lozano-Sánchez J., Beltrán-Debón R., Joven J., Segura-Carretero A., Fernádez-Gutierrez A. (2013). Phenolic characterization and geographical classification of commercial Arbequina extra-virgin olive oils produced in southern Catalonia. Food Res. Int..

[26] Gargouri B., Ammar S., Zribi A., Mansour A., Bouaziz M. (2013). Effect of growing region on quality characteristics and phenolic compounds of Chemlali extra-virgin olive oils. Acta Physiol. Plant..

[27] Ben Hlima H., Ben Ayed R., Ennouri K., Smaoui S. (2017). Geographical discrimination of virgin olive oils from the tunisian coasts by combining fatty acids and phenolic acids profiles within a multivariate analysis. J. Oleo Sci..

[28] Criado M.N., Morelló J.R., Motilva M.J., Romero M.P. (2004). Effect of growing area on pigment and phenolic fractions of virgin olive oils of the arbequina variety in Spain. J. Am. Oil Chem. Soc..

[29] Becerra-Herrera M., Vélez-Martín A., Ramos-Merchante A., Richter P., Beltrán R., Sayago A. (2018). Characterization and evaluation of phenolic profiles and color as potential discriminating features among Spanish extra virgin olive oils with protected designation of origin. Food Chem..

[30] Dugo L., Russo M., Cacciola F., Mandolfino F., Salafia F., Vilmercati A., Fanali C., Casale M., de Gara L., Dugo P. (2020). Determination of the phenol and tocopherol content in italian high-quality extra-virgin olive oils by using LC-MS and multivariate data analysis. Food Anal. Methods.

[31] Klikarová J., Česlová L., Kalendová P., Dugo P., Mondello L., Cacciola F. (2020). Evaluation of Italian extra virgin olive oils based on the phenolic compounds composition using multivariate statistical methods. Eur. Food Res. Technol..

[32] Fanali C., Della Posta S., Vilmercati A., Dugo L., Russo M., Petitti T., Mondello L., de Gara L. (2018). Extraction, analysis, and antioxidant activity evaluation of phenolic compounds in different italian extra-virgin olive oils. Molecules.

[33] Baiano A., Terracone C., Viggiani I., Del Nobile M.A. (2013). Effects of cultivars and location on quality, phenolic content and antioxidant activity of extra-virgin olive oils. J. Am. Oil Chem. Soc..

[34] Taamalli A., Arráez Román D., Zarrouk M., Segura-Carretero A., Fernández-Gutiérrez A. (2012). Classification of ‘Chemlali’ accessions according to the geographical area using chemometric methods of phenolic profiles analysed by HPLC–ESI-TOF–MS. Food Chem..

[35] Alkan D., Tokatli F., Ozen B. (2012). Phenolic characterization and geographical classification of commercial extra virgin olive oils produced in Turkey. J. Am. Oil Chem. Soc..

[36] Kesen S., Kelebek H., Selli S. (2014). LC-ESI-MS characterization of phenolic profiles Turkish olive oils as influenced by geographic origin and harvest year. J. Am. Oil Chem. Soc..

[37] Petrakis P.V., Agiomyrgianaki A., Christophoridou S., Spyros A., Dais P. (2008). Geographical characterization of Greek virgin olive oils (Cv. Koroneiki) using 1H and 31P NMR fingerprinting with canonical discriminant analysis and classification binary trees. J. Agric. Food Chem..

[38] Kosma I., Vatavali K., Kontakos S., Kontominas M., Kiritsakis A., Badeka A. (2017). Geographical differentiation of Greek extra virgin olive oil from late-harvested koroneiki cultivar fruits. J. Am. Oil Chem. Soc..

[39] Lukic I., Luki M., Žanetic M., Krapac M., Godena S., Brkic Bubola K. (2019). Inter-Varietal diversity of typical volatile and phenolic profiles of croatian extra virgin olive oils as revealed by GC-IT-MS and UPLC-DAD analysis. Foods.

[40] Mansouri F., Ben Moumen A., Aazza S., Belhaj K., Fauconnier M.L., Sindic M., Serghini Caid H., Elamrani A. (2019). Quality and chemical profiles of virgin olive oils of three European cultivars suitable for super-high-density planting conditions in eastern Morocco. Mater. Today Proc..

[41] Tasioula-Margari M., Tsabolatidou E. (2015). Extraction, separation, and identification of phenolic compounds in virgin olive oil by HPLC-DAD and HPLC-MS. Antioxidants.

[42] Kalogiouri N.P., Alygizakis N.A., Aalizadeh R., Thomaidis N.S. (2016). Olive oil authenticity studies by target and nontarget LC–QTOF-MS combined with advanced chemometric techniques. Anal. Bioanal. Chem..

[43] Alarcón Flores M.I., Romero-González R., Garrido Frenich A., Martínez Vidal J.L. (2012). Analysis of phenolic compounds in olive oil by solid-phase extraction and ultra high performance liquid chromatography–tandem mass spectrometry. Food Chem..

[44] Kıvrak Ş., Kıvrak İ. (2020). Ultrasonic-assisted extraction method of phenolic compounds in Extra-Virgin Olive Oils (EVOOs) by Ultra Performance Liquid Chromatography–Tandem Mass Spectrometry (UPLC–MS/MS). Sep. Sci. Technol..

[45] Kalogiouri N.P., Aalizadeh R., Dasenaki M.E., Thomaidis N.S. (2020). Application of high resolution mass spectrometric methods coupled with chemometric techniques in olive oil authenticity studies—A review. Anal. Chim. Acta.

[46] Jerman Klen T., Golc Wondra A., Vrhovšek U., Mozetič Vodopivec B. (2015). Phenolic profiling of olives and olive oil process-derived matrices using UPLC-DAD-ESI-QTOF-HRMS analysis. J. Agric. Food Chem..

[47] Kalogiouri N.P., Aalizadeh R., Thomaidis N.S. (2018). Application of an advanced and wide scope non-target screening work fl ow with LC-ESI-QTOF-MS and chemometrics for the classification of the Greek olive oil varieties. Food Chem..

[48] Kalogiouri N.P., Aalizadeh R., Dasenaki M.E., Thomaidis N.S. (2020). Authentication of Greek PDO kalamata table olives: A novel non-target high resolution mass spectrometric approach. Molecules.

[49] Kritikou E., Kalogiouri N.P., Kolyvira L., Thomaidis N.S. (2020). Target and suspect HRMS metabolomics for the 13 varieties of olive leaves and drupes from Greece. Molecules.

[50] Kalogiouri N.P., Aalizadeh R., Thomaidis N.S. (2017). Investigating the organic and conventional production type of olive oil with target and suspect screening by LC-QTOF-MS, a novel semi-quantification method using chemical similarity and advanced chemometrics. Anal. Bioanal. Chem..

[51] Michel T., Khlif I., Kanakis P., Termentzi A., Allouche N., Halabalaki M., Skaltsounis A.-L. (2015). UHPLC-DAD-FLD and UHPLC-HRMS/MS based metabolic profiling and characterization of different *Olea europaea* organs of Koroneiki and Chetoui varieties. Phytochem. Lett..

[52] Tóth G., Alberti Á., Sólyomváry A., Barabás C., Boldizsár I., Noszál B. (2015). Phenolic profiling of various olive bark-types and leaves: HPLC-ESI/MS study. Ind. Crops Prod..

[53] Kanakis P., Termentzi A., Michel T., Gikas E., Halabalaki M., Skaltsounis A.-L. (2013). From olive drupes to olive Oil. An HPLC-orbitrap-based qualitative and quantitative exploration of olive key metabolites. Planta Med..

[54] Lozano-Sanchez J., Segura-Carretero A., Menendez J.A., Oliveras-Ferraros C., Cerretani L., Fernandez-Gutierrez A. (2010). Prediction of extra virgin olive oil varieties through their phenolic profile. potential cytotoxic activity against human breast cancer cells. J. Agric. Food Chem..

[55] Dierkes G., Krieger S., Duck R., Bongartz A., Schmitz O.J., Hayen H. (2012). High-performance liquid chromatography−mass spectrometry profiling of phenolic compounds for evaluation of olive oil bitterness and pungency. J. Agric. Food Chem..

[56] Capriotti A.L., Cavaliere C., Crescenzi C., Foglia P., Nescatelli R., Samperi R., Laganà A. (2014). Comparison of extraction methods for the identification and quantification of polyphenols in virgin olive oil by ultra-HPLC-QToF mass spectrometry. Food Chem..

[57] Lopez-Yerena A., Ninot A., Jimenez-Ruiz N., Lozano-Castellon J., Perez M., Escribano-Ferrer E., Romero-Aroca A., Lamuela-Raventos R., Vallverdu-Queralt A. (2021). Influence of the ripening stage and extraction conditions on the phenolic fingerprint of ‘corbella’ extra-virgin olive oil. Antioxidants.

[58] Klikarová J., Rotondo A., Cacciola F., Ceslova L., Dugo P., Mondello L., Rigano F. (2019). The phenolic fraction of Italian extra virgin olive oils: Elucidation through combined liquid chromatography and NMR approaches. Food Anal. Methods.

[59] Rozanska A., Russo M., Cacciola F., Salafia F., Polkowska Z., Dugo P., Mondello L. (2020). Concentration of potentially bioactive compounds in italian extra virgin olive oils from various sources by using LC-MS and multivariate data analysis. Foods.

[60] Diamantakos P., Ioannidis K., Papanikolaou C., Tsolakou A., Rigakou A., Melliou E., Magiatis P. (2021). A new definition of the term “high-phenolic olive oil” based on large scale statistical data of Greek olive oils analyzed by qNMR. Molecules.

[61] Loubiri A., Taamalli A., Talhaoui N., Mohamed S.N., Segura-Carretero A., Zarrouk M. (2017). Usefulness of phenolic profile in the classification of extra virgin olive oils from autochthonous and introduced cultivars in Tunisia. Eur. Food Res. Technol..

[62] Bajoub A., Ajal E.A., Fernandez-Gutierrez A., Carrasco-Pancorbo A. (2016). Evaluating the potential of phenolic profiles as discriminant features among extra virgin olive oils from Moroccan controlled designations of origin. Food Res. Int..

[63] El Hilali H., El Hilali F., Porter S.E.G., Ghali S.A., Meyls H.M., Ouazzani N., Laziri F., Barber A. (2020). Olive oil varieties cultivated in Morocco reduce reactive oxygen species and cell viability of human cervical cancer cells. Med. J. Nutr. Metab..

[64] Kelebek H., Kesen S., Selli S. (2015). Comparative study of bioactive constituents in Turkish olive oils by LC-ESI/MS/MS. Int. J. Food Prop..

[65] García-Rodríguez R., Belaj A., Romero-Segura C., Sanz C., Pérez A.G. (2017). Exploration of genetic resources to improve the functional quality of virgin olive oil. J. Funct. Foods.

[66] Gambacorta G., Faccia M., Trani A., Lamacchia C., Gomes T. (2012). Phenolic composition and antioxidant activity of Southern Italian monovarietal virgin olive oils. Eur. J. Lipid Sci. Technol..

[67] Cioffi G., Pesca M.S., De Caprariis P., Braca A., Severino L., De Tommasi N. (2010). Phenolic compounds in olive oil and olive pomace from Cilento (Campania, Italy) and their antioxidant activity. Food Chem..

