# Supplementary material for: Geographical Characterization of Olive Oils from the North Aegean Region Based on the Analysis of Biophenols with UHPLC-QTOF-MS
Source: Foods. 2021 Sep 6;10(9):2102. doi: 10.3390/foods10092102 (PMC8468971; doi:10.3390/foods10092102)
Supplement: Supplementary file 1 [file foods-10-02102-s001.zip › foods-1327639-supplementary.pdf]

Supplementary Material

Geographical Characterization of Olive Oils from the North Aegean Region Based on the Analysis of Biophenols with UHPLC-QTOF-MS

Evangelia Kritikou, Natasa P. Kalogiouri, Marios Kostakis, Dimitrios–Christos Kanakis, Ioannis Martakos, Constantina Lazarou, Michalis Pentogennis and Nikolaos S. Thomaidis *

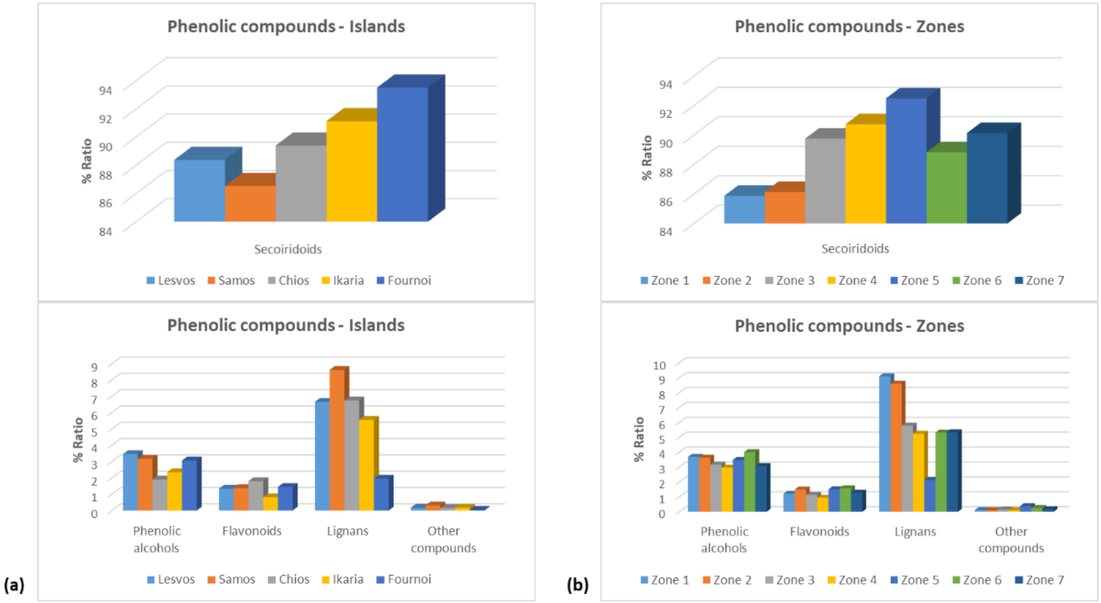

Figure S1. Types of phenolic compounds (%) in EVOOs between the (**a**) islands; (**b**) zones of Lesvos.

Table S1. Gradient program of mobile phase.

t_R (min)	Flow (μL/min)	%A
0.0	200	1.0
1.0	200	1.0
3.0	200	39.0
14.0	400	99.9
16.0	480	99.9
16.1	200	1.0
20.0	200	1.0

Table S2. The main olive tree varieties and the number of EVOOs samples in the five islands of the North Aegean Region.

Island	Olive tree varieties	Number of samples
Lesvos	Kolovi, Adramitiani, Koroneiki, Agrilia, Ladolia	363
Samos	Koroneiki, Throumpa, Manaki	51
Chios	Koroneiki, Throumpa, Chiotiki (native), Dafnelia	20
Ikaria	Koroneiki, Chondrolia	12
Fournoi	Koroneiki, Manaki	6

Table S3. Number of samples in each geographical zone of the island of Lesvos.

Zone	Number of samples
1	30
2	112
3	59
4	20
5	14
6	49
7	64

Table S4. QC results.

Compound	%RSD of Peak Area, $n = 12$	%RSD of t_R (min), $n = 12$	Δm (±error, mDa), $n = 12$
Apigenin	2.35	0.06	-0.05
Hydroxytyrosol	4.50	0.09	0.15
Oleuropein	2.72	0.04	-0.18
Tyrosol	5.02	0.07	-0.06
Vanillin	4.08	0.11	0.04

Table S5. List of target compounds [1].

Compound	Molecular Formula	*m/z* [M−H]⁻ Theoretical	t_R Standard (min)	Fragments *m/z*	Elemental Formula
		**Phenolic Acids**			
Caffeic acid	$C_9H_8O_4$	179.0349	1.53	-	-
Ferulic acid	$C_{10}H_{10}O_4$	193.0506	1.40	134.0370	$C_8H_6O_2$
				178.0271	$C_9H_6O_4$
Gallic acid	$C_7H_6O_5$	169.0142	1.25	125.0246	$C_6H_5O_3$
Homovanillic acid	$C_9H_{10}O_4$	181.0506	1.50	59.0134	$C_2H_3O_2$
				122.0369	$C_7H_6O_2$
				137.0610	$C_8H_9O_2$
				154.0266	$C_7H_6O_4$
p-Coumaric acid	$C_9H_8O_3$	163.0400	1.34	93.0349	C_6H_5O
				119.0506	C_8H_7O
Syringic acid	$C_9H_{10}O_5$	197.0455	1.44	182.0218	$C_8H_6O_5$
		**Phenolic Alcohols**			
Hydroxytyrosol	$C_8H_{10}O_3$	153.0557	3.53	123.0446	$C_7H_7O_2$
Tyrosol	$C_8H_{10}O_2$	137.0608	4.07	81.0262	C_5H_5O
				93.0345	C_6H_5O
				112.0530	$C_6H_8O_2$
		**Phenolic Aldehydes**			
Vanillin	$C_8H_8O_3$	151.0400	4.73	71.0140	$C_3H_3O_2$
				95.0140	$C_5H_3O_2$
				108.0217	$C_6H_4O_2$
				136.0162	$C_7H_4O_3$
		**Flavonoids**			
Apigenin	$C_{15}H_{10}O_5$	269.0455	8.24	149.0248	$C_8H_5O_3$
				151.0037	$C_7H_3O_4$
Epicatechin	$C_{15}H_{14}O_6$	289.0716	4.37	137.0248	$C_7H_5O_3$
				151.0416	$C_8H_7O_3$
Luteolin	$C_{15}H_{10}O_6$	285.0404	7.55	133.0295	$C_8H_5O_2$
				151.0036	$C_7H_3O_4$
		**Secoiridoids**			
Oleuropein	$C_{25}H_{32}O_{13}$	539.1770	5.96	59.0138	$C_2H_3O_2$
				89.0244	$C_3H_5O_3$
				101.0242	$C_4H_5O_3$
				111.0083	$C_5H_3O_3$
				121.0295	$C_7H_5O_2$
				307.0823	$C_{15}H_{15}O_7$
				377.1242	$C_{19}H_{21}O_8$
		**Lignans**			
Pinoresinol	$C_{20}H_{22}O_6$	357.1343	6.49	151.0399	$C_8H_7O_3$

Table S6. List of suspect compounds [1].

Compound	Molecular Formula	*m/z* [M−H]⁻ Calculated	Experimental t_R (min)	Predicted t_R (min)	Fragments *m/z*	Elemental Formula
			**Phenolic Alcohols**			
Hydroxytyrosol acetate	$C_{10}H_{12}O_4$	195.0663	6.71	6.48	134.0373	$C_8H_6O_2$
					149.0608	$C_9H_9O_2$
					161.0246	$C_9H_5O_3$
			**Secoiridoids**			
Decarboxymethyl ligstroside aglycone (Oleocanthal)	$C_{17}H_{20}O_5$	303.1237	6.42	6.76	124.0531	$C_7H_8O_2$
					137.0608	$C_8H_{10}O_2$
					147.0453	$C_9H_7O_2$
					165.0556	$C_9H_9O_3$
					183.0662	$C_9H_{11}O_4$
Decarboxymethyl oleuropein aglycone (Oleacein)	$C_{17}H_{20}O_6$	319.1187	5.61	6.14	69.0342	C_4H_5O
					95.0502	C_6H_7O
					123.0451	$C_7H_7O_2$
					139.0608	$C_8H_{11}O_2$
					165.0556	$C_9H_9O_3$
					183.0660	$C_9H_{11}O_4$
					195.0656	$C_{10}H_{11}O_4$
10-Hydroxy-10-methyl oleuropein aglycone	$C_{20}H_{24}O_9$	407.1347	6.71	6.75	99.0453	$C_5H_7O_2$
					111.0087	$C_5H_3O_3$
					121.0295	$C_7H_5O_2$
					135.0453	$C_8H_7O_2$
					137.0243	$C_7H_5O_3$
					149.0245	$C_8H_5O_3$
					163.0402	$C_9H_7O_3$
					179.0351	$C_9H_7O_4$
					195.0665	$C_{10}H_{11}O_4$
					241.0871	$C_{15}H_{13}O_3$
10-Hydroxy decarboxymethyl oleuropein aglycone	$C_{17}H_{20}O_7$	335.1136	4.28	5.52	59.0139	$C_2H_3O_2$
					85.0296	$C_4H_5O_2$
					121.0292	$C_7H_5O_2$
					151.0401	$C_8H_7O_3$
					153.0557	$C_8H_9O_3$
					155.0716	$C_8H_{11}O_3$
					199.0613	$C_9H_{11}O_5$
10-Hydroxy oleuropein aglycone	$C_{19}H_{22}O_9$	393.1191	4.82	5.48	137.0244	$C_7H_5O_3$
					181.0502	$C_9H_9O_4$
Ligstroside aglycone	$C_{19}H_{22}O_7$	361.1292	6.63	6.83	259.0975	$C_{15}H_{15}O_4$
					291.0875	$C_{15}H_{15}O_6$
Methyl oleuropein aglycone	$C_{20}H_{24}O_8$	391.1398	7.51	7.37	59.0140	$C_2H_3O_2$
					67.0192	C_4H_3O
					99.0456	$C_5H_7O_2$
					111.0087	$C_5H_3O_3$
					137.0608	$C_8H_9O_2$
					291.0875	$C_{16}H_{15}O_6$
Oleoside	$C_{16}H_{22}O_{11}$	389.1089	7.91	[1]	113.0244	$C_5H_5O_3$
					139.0032	$C_6H_3O_4$
					149.0240	$C_8H_5O_3$

Compound	Molecular Formula	*m/z* [M−H]⁻ Calculated	Experimental t_R (min)	Predicted t_R (min)	Fragments *m/z*	Elemental Formula
					165.0552	$C_9H_9O_3$
					183.0666	$C_9H_{11}O_4$
Oleuropein aglycone	$C_{19}H_{22}O_8$	377.1241	7.29	6.88	$C_5H_3O_3$	$C_5H_3O_3$
					$C_8H_5O_3$	$C_8H_5O_3$
					$C_{10}H_{11}O_4$	$C_{10}H_{11}O_4$
					$C_{15}H_{15}O_5$	$C_{15}H_{15}O_5$
					$C_{15}H_{15}O_7$	$C_{15}H_{15}O_7$
			**Lignans**			
1-Acetoxypinoresinol	$C_{22}H_{24}O_8$	415.1398	6.42	7.20	151.0402	$C_8H_7O_3$
					280.0951	$C_{14}H_{16}O_6$
					343.1188	$C_{19}H_{19}O_6$
1-Hydroxypinoresinol	$C_{20}H_{22}O_7$	373.1292	6.39	6.39	121.0294	$C_7H_5O_2$
					151.0401	$C_8H_7O_3$
					163.0402	$C_9H_7O_3$
Syringaresinol	$C_{22}H_{26}O_8$	417.1554	6.18	8.10	127.0408	$C_6H_7O_3$
					181.0503	$C_9H_9O_4$
			**Other compounds**			
Elenolic acid	$C_{11}H_{14}O_6$	241.0717	4.51	4.26	59.0137	$C_2H_3O_2$
					95.0496	C_6H_7O
					127.0400	$C_6H_7O_3$
					151.0402	$C_8H_7O_3$
					171.0300	$C_7H_7O_5$
Hydroxylated form of elenolic acid	$C_{11}H_{14}O_7$	257.0667	1.36	[1]	59.0104	$C_2H_3O_2$
					137.0603	$C_8H_9O_2$
					181.0535	$C_9H_9O_4$

[1] The retention time prediction results are not reliable and other methods of verification such as the tandem mass spectrometry fragmentation pattern should be applied.

Table S7. Standard calibration curve and coefficient of determination (r^2) for tyrosol, hydroxytyrosol, oleuropein, apigenin, luteolin and pinoresinol.

Compound	Calibration Curve $y = (b \pm S_b)\,x + (a \pm S_a)$	r^2
Tyrosol	y = (0.408 ± 0.010) x – (0.092 ± 0.059)	0.998
Hydroxytyrosol	y = (3.411 ± 0.034) x – (0.24 ± 0.21)	0.9996
Oleuropein	y = (1.122 ± 0.019) x – (0.15 ± 0.12)	0.999
Apigenin	y = (8.41 ± 0.27) x + (17.3 ± 1.7)	0.996
Luteolin	y = (5.63 ± 0.16) x – (1.50 ± 0.97)	0.997
Pinoresinol	y = (0.1652 ± 0.0044) x – (0.055 ± 0.027)	0.997

References

1. Kalogiouri, N.P.; Alygizakis, N.A.; Aalizadeh, R.; Thomaidis, N.S. Olive oil authenticity studies by target and nontarget LC–QTOF-MS combined with advanced chemometric techniques. *Anal. Bioanal. Chem.* **2016**, *408*, 7955–7970, doi:10.1007/s00216-016-9891-3.
